# A Biomimetic Polymer for the Extraction and Purification of Superior Analogues of Amphotericin B

**DOI:** 10.3390/biomimetics8030273

**Published:** 2023-06-27

**Authors:** Todd Cowen, Simon Walmsley, Kal Karim, Resul Haser, Patrick Caffrey, Elena Piletska, Bernard Rawlings, Sergey A. Piletsky

**Affiliations:** 1 School of Chemistry, University of Leicester, Leicester LE1 7RH, UK; 2 School of Biomolecular and Biomedical Science, University College Dublin, D04 V1W8 Dublin, Ireland

**Keywords:** amphotericin B, ligand design, biomimetic polymers, leishmaniasis, molecularly imprinted polymers

## Abstract

Amphotericin B has been an essential drug in the fight against leishmaniasis and fungal pathogens for decades, and has more recently gained attention for the very limited microbial resistance displayed against it. However, its toxicity has restricted its use to only the most severe cases of disease, and attempts to reduce these ill effects via formulation have had only minor success. Genetic engineering has allowed the development of superior amphotericin analogues, notably 16-descarboxyl-16-methyl amphotericin B (MeAmB), which shows a ten-fold reduction in toxicity in addition to a slight improvement in therapeutic activity. However, MeAmB is difficult to extract from its bacterial source and purify. Presented here is an alternative method of MeAmB purification. A biomimetic polymer with a high affinity for MeAmB was designed via computational modelling and synthesised. Prepared as a separation column, the polymer was able to retain the target MeAmB whilst allowing the removal of cell debris from the bacterial extract. Starting with a simple bacterial extract, the relatively simple process allowed the purification of an MeAmB salt complex at approximately 70% MeAmB, and likely higher purification from further extraction. The mean MeAmB recovery between the pre-purification extract sample and the final product was 81%. This is the first successful demonstration of extraction or purification of any amphotericin molecule with any polymeric material. The biomimetic polymer was additionally reusable and simple to fabricate, giving this technique significant advantages over traditional methods of extraction and purification of valuable compounds.

## 1. Introduction

The properties of amphotericin B (AmB) were first described in 1956 after its isolation from a species of *Streptomyces*, later named *S. nodosus*, found in soil samples taken from Temblador in the Oninoco river basin of Venezuela [1]. AmB rapidly became the gold standard in the treatment of fungal infections, and retained this status for decades [2]. AmB is also routinely applied in cases of leishmaniasis, being the primary medication for leishmaniasis cases with immunocompromised patients [3]. AmB is, however, associated with nephrotoxicity, which has encouraged the search for more suitable formulations and analogues [4].

**Figure 1:**
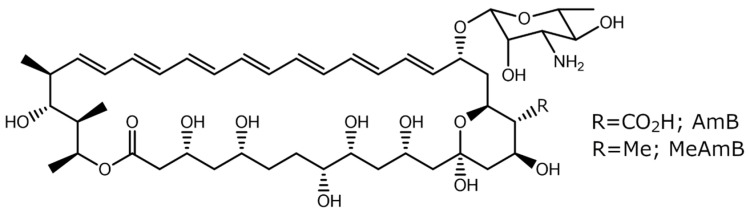
Amphotericin B (AmB) and 16-descarboxyl-16-methyl-amphotericin B (MeAmB).

16-descarboxyl-16-methyl-amphotericin B (MeAmB) is an AmB analogue with a methyl in place of the AmB carboxyl (Figure 1). Synthesis of MeAmB is relatively simple to induce via genetic modification of *S. nodosus*, as oxidation of the methyl to carboxyl is a post-polyketide synthase process which can be inhibited [5,6]. The genetically modified *S. nodosus* strain also produces a tetraene co-metabolite, 8-deoxy-16-descarboxyl-16-methyl-amphotericin A (MeAmA) [6]. In a study of the efficacy of MeAmB in preventing Candida albicans infection, the observed minimum inhibitory concentration (MIC) of the new compound was 1 µg cm^−3^, giving it slightly greater activity than conventional AmB (which has an MIC of 1.25 µg cm^−3^). Of more interest, however, was the reduced toxicity of MeAmB, which displayed a minimum homolytic concentration (MHC) of 50 µg cm^−3^, an order of magnitude higher than that of AmB (5 µg cm^−3^) [6].

Wild *S. nodosus* can produce AmB at levels of 4 g dm^−3^ in simple, low-cost fermentation processes [5]. The compound is extracted from the culture using alcohol, with substantial amounts of bacterial cell debris (principally membrane lipids and polysaccharides) resulting in a mixture of approximately 1% target compound. Patient chromatographic methods and repeated solvent extraction can produce a useful product, but the resources involved greatly affect the final market value.

Conventional methods, therefore, provide a low overall yield of the drug and require large quantities of organic solvents. This arises primarily due to the low but consistent solubility and affinity of amphotericin B for most common solvents and chromatographic phases. This problem is exacerbated with amphotericin B analogues such as MeAmB, which are produced in much lower concentrations. What is required, therefore, is a technique which selectively binds to the target compound while allowing all other material to be washed away, followed by the extraction of the purified target.

Herein, we describe the development and application of a biomimetic, polymeric material developed for the separation of MeAmB from bacterial media. Inspired by natural antibodies and molecularly imprinted polymers [7], the material was computationally optimised using a reverse ligand design protocol to optimise binding to the specific target molecule. The resulting material allows a purification process which is simple and efficient, resulting in a practical improvement in the extraction of amphotericin compounds. This should provide a foundation for the large-scale extraction of MeAmB and similar analogues, providing more readily available treatments for leishmaniasis and other infections.

## 2. Materials and Methods

All reagents were obtained from Sigma-Aldrich or its subsidiaries. The bacterial cultures were prepared using a Priorclave autoclave and solvent extractions of products involved the use of a Centurion 1000 series centrifuge running at 4530 rpm. Analysis was performed using a Shimadzu UV-2401PC spectrophotometer, a Varian ProStar HPLC system and a Bruker Avance DPX 300 MHz NMR spectrometer. HPLC was performed as a reverse phase method using methanol and water, with the ratio between the two initially being 1:1 and increasing gradually over 35 min to a 100% methanol mobile phase. A Labconco FreeZone 2.5 lyophiliser was regularly used in the preparation of samples. In silico molecular modelling was performed using the software package Sybyl 7.3 (Tripos Inc., St. Louis, MO, USA) incorporating the Leapfrog algorithm.

### 2.1. 16-Descarboxyl-16-methyl Amphotericin B (MeAmB) Production

The *S. nodosus* NM mutant was developed via a homologous recombination gene replacement strategy. The genome of the wild-type *S. nodosus* was altered via targeted removal of the amphN gene, which codes for the P450 monooxygenase AmphN enzyme, and its facilitating amphM ferredoxin gene [5].

The *S. nodosus* NM mutant was stored at approximately −80 °C in microcentrifuge tubes, each of which was used to inoculate one 100 cm^3^ sample of inoculation media. The inoculation media was produced from glucose (1 g), yeast extract (1 g), deionised water (100 cm^3^) and sodium hydroxide solution (2 M, approximately 150 μL), which were combined in 500 cm^3^ tri-grooved flasks and autoclaved at 130 °C for 3 h. The inoculation media were left for at least 1 week before the contents were transferred to each of 8 production media flasks, with each acquiring 10 cm^3^ of inoculation culture. The production media was composed of a similarly autoclaved mixture of fructose (5 g), dextrin (15 g) soybean flour (7.5 g, unroasted), calcium carbonate (2.5 g), Amberlite XAD16N styrene-divinylbenzene adsorbent (10 g) and deionised water (250 cm^3^). The production flasks were incubated for approximately one week, after which they were drained of aqueous liquid and contained in a closed cotton fabric.

The drained sample was then submerged in methanol, which was replaced daily. The MeAmB content of the methanolic extract was measured using spectrophotometry. Both the AmB and AmA analogues had been studied previously and molar absorption coefficients were determined, with heptaenes giving ε of 1.7 × 10^5^ M^−1^ cm^−1^ at 405 nm and tetraenes ε of 0.78 × 10^5^ M^−1^ cm^−1^ at 318 nm [5]. A characteristic three-peak absorption pattern was observed for amphotericin B and its analogues, and the quantity of heptaene (MeAmB) present could be determined by rearranging the Beer–Lambert law and applying it to the peak corresponding to the longest wavelength:

$$mass\left(mg\right)=\frac{\left[abs.405\mathrm{nm}\right]\times total\;volume\left(\mathrm{mL}\right)\times dilution}{\left(\epsilon =200\right)\times (l=1\mathrm{cm})}$$

A similar equation was used to determine the quantity of tetraene MeAmA present in the sample:

$$mass\left(mg\right)=\frac{\left[abs.319-325\mathrm{nm}\right]\times total\;volume\left(\mathrm{mL}\right)\times dilution}{\left(\epsilon =100)\times (l=1\mathrm{cm}\right)}$$

An average of just over 100 mg of MeAmB was obtained per litre of production medium. Samples were prepared for further analysis through the solvent extraction of crude bacterial precipitate, initially obtained through the evaporation of the crude methanol extract. Deionised water was added to the solid and sonicated to form a suspension, then centrifuged repeatedly until the water appeared clear. This process was then repeated using diethyl ether and ethyl acetate. A product of approximately 1% purity resulted. Repetition of this process gave higher purity samples, but with smaller improvements in purity and approximately constant loss of MeAmB in each repetition.

### 2.2. Computational Modelling

The target 16-descarboxyl-16-methyl amphotericin B (MeAmB) was constructed in silico and energetically minimised to the global minimum via simulated annealing, with periodic molecular dynamics and minimisation being performed in stages between 600 K and 300 K. A pre-minimised library of 40 monomers was screened against the MeAmB target using the Leapfrog ligand design algorithm. The program calculated optimal interaction sites around the target via the approximation of the electronic density surrounding the MeAmB. Taking these sites as starting points, the program efficiently trialed each monomer in each site, optimising with small movements and geometric rearrangements of the monomer. The binding energies found between the target and the monomers at the end of this process ere ranked, providing an association energy and a geometry of that interaction. The optimal ratio of monomers was then determined according to the site of monomer interaction with the target and interaction between the individual monomers. An optimal synthetic receptor was, therefore, built from the monomers for the MeAmB analyte.

### 2.3. Polymer Synthesis and Selection

Monomer combinations were prepared in the ratio prescribed by the design software. Briefly, 10 g of the composition was solvated in 10 g of dimethylformamide (DMF) with 100 mg of radical polymerisation initiator 1,1′-azobis(cyclohexanecarbonitrile) (ABCN). The mixture was flushed with nitrogen and heated under an inert atmosphere at 80 °C for 12 h. The resulting polymer was then ground and sieved to give particles of approximately 35 µm to 125 µm based on the specifications of the equipment. The desired masses of each polymer were then inserted into polypropylene columns fitted with polyethylene filters.

Two 100 mg columns of each polymer were tested with a standard sample of approximately 10 µg cm^−3^ MeAmB. Crude methanolic extract of an *S. nodosus* culture (concentrated without purification, 1 cm^3^) was added to each column and allowed to pass through the polymer at a rate of approximately one drop every two seconds. The resulting samples collected from the vacuum chamber were analysed using spectrophotometry to determine the quantity of MeAmB retained.

Each column was then washed with 2 cm^3^ of water, 3 cm^3^ of basic (pH 9) aqueous solution of sodium hydrogen carbonate, 2 cm^3^ of methanol and 2 cm^3^ of ethyl acetate. In polymer A (5% ethylene glycol methacrylate phosphate, EGMP), no heptaene (MeAmB) or tetraene (MeAmA) was detected in the water or NaHCO_3_ washes. The first equivalent of methanol removed 1.2 µg (15% of column contents, 12% of total starting sample) of MeAmB, but nothing was present in the second. On repetition with a material synthesised from 10% TFMA and 90% EGDMA (polymer H), more MeAmB was found in the methanol elution, and this polymer was selected for the remainder of the project.

### 2.4. Small-Scale Protocol Optimisation for the Extraction of 16-Descarboxyl-16-methyl Amphotericin B (MeAmB)

Samples were added to the polymer column in a methanol solution. It was noted that the selected polymer was slightly unstable in methanol, initially giving small polymer particles in the eluate in a manner that was not observed with other solvents or noted with other polymers. Detrimental effects of this were overcome by conditioning the polymer with methanol prior to use, which stopped these particles appearing in the product.

Two columns of 100 mg of polymer were loaded with 4 sequential additions of 1 mL sample at 156.7 μg cm^−3^ MeAmB (1.6% purity) to give an average total retention of 261.7 μg per column. Each column was then washed with 3 cm^3^ of water and 3 cm^3^ of dichloromethane. From spectrophotometric analysis of the dichloromethane, no observable MeAmB was removed in this process, while the water sample contained approximately 1.5 µg of MeAmB. The water wash removed 0.35 mg of impurity per column, and the dichloromethane wash gave yellow/orange material with a mass of 3.1 mg. Results were more dramatic with samples that had less pre-purification; for example, the washing of two 71.5 µg cm^−3^ MeAmB samples with water and dichloromethane resulted in the water removing 2.4 mg of impurities from each polymer and no MeAmB, while the dichloromethane removed 6.1 mg per column including no product MeAmB.

Washing with hexane, a possible solvent for some of the impurities, resulted in no eluted material, product or cell debris. Diethyl ether removed 3.2 mg of impurities, identified as lipids and amphotericin A by NMR, but following the ether wash with ethyl acetate removed an additional 16 mg of lipids from the column

A ‘Folch mix’ was formed with chloroform, methanol and water mixed in a ratio of 8:4:3. This formed two layers, the denser of which had a ratio of approximately 86:14:1 (chloroform/methanol/water), and was used as a final wash of the polymer. This was found to be very effective at removing impurities from the column, but also removed approximately 11% of the bound MeAmB which was initially bound to the polymer. This was considered an excessive loss in product, and the Folch mix was not pursued further. The methanol content of the mix apparently promoted MeAmB removal, an effect which was also noted with other alcohols.

MeAmB retention was studied with two 100 mg polymer columns, each conditioned with 2 cm^3^ of water and 3 cm^3^ of methanol. Briefly, 4 cm^3^ of a 72 µg cm^−3^ MeAmB sample were passed through each column, with each millilitre being collected separately for analysis. Extractions were attempted with DMSO, but this was found to be unsuitable.

For MeAmB extraction, 6 cm^3^ of bacterial extract was passed through two columns of 100 mg polymer for a total retention of 309 µg. After losing approximately 58 μg of MeAmB in washing, the remaining product was eluted with 3 cm^3^ of 2 M methanol solution of ammonium formate. The first eluate contained 216 µg of MeAmB, the second contained 8.6 µg and the third 5.6 µg.

Selectivity of the polymer for the target was then tested with a partially purified sample (3% MeAmB by solid mass), which contained 108.4 µg cm^−3^ MeAmB and 101.7 µg cm^−3^ MeAmA (tetraene). A total of 4 cm^3^ of sample was passed through each of two columns, but with each millilitre being run three times.

### 2.5. Large-Scale MeAmB Purification and Analysis

A single 3 g polymer was prepared via thorough conditioning with water and methanol. An initial sample (248 µg cm^−3^, 11% purity) was run through the column in three 22.5 cm^3^ volumes and washed using the previously described protocol. Extraction was performed with two 15 cm^3^ volumes of 2 M methanolic solution of ammonium formate. The first MeAmB extract produced an absorption spectrum equivalent to 5.84 mg, and the second 0.21 mg. The product was combined in a small flask and the methanol was removed. A total of 1.5 cm^3^ of deionised water was added to the solid product, which was then filtered, and the residue was collected. From a loaded MeAmB content of 4.320 mg, the aqueous media with dissolved ammonium formate contained approximately 20 µg of MeAmB. The product was estimated to contain 21.4% MeAmB (13.0 mg total mass) and was of sufficient quantity for NMR analysis. Assignment of the peaks was aided by the work of Sowinski et al. [8]; more details are given in Supplementary Section S2.

NMR: δ_H_(300 MHz; CD_3_OD; Me_4_Si) 0.75 (3 H, d, *J* 10.7, C^34^-C*H*_3_), 0.80 (3 H, d, *J* 8.1, C^16^-C*H*_3_), 0.91 (3 H, d, *J* 7.2, C^36^-C*H*_3_), 1.02 (3 H, d, *J* 6.7, C^37^-C*H*_3_), 1.10 (3 H, d, *J* 6.5, C^5′^-C*H*_3_), 1.20–1.80 (7 H, m, C^4^*H*_2_, C^6^*H*, C^10^*H*, C^12^*H*, C^14^*H*, C^16^*H*), 1.80–2.50 (9 H, m, C^2^*H*_2_, C^3^*H*, C^6^*H*, C^7^*H*, C^10^*H*, C^14^*H*, C^18^*H*, C^34^*H*), 2.90–3.60 (4 H, m, C^8^*H*, C^18^*H*, C^35^*H*, C^3′^*H*), 3.82 (2 H, m, C^9^*H*, C^5′^*H*), 4.23 (2H, m, C^5^*H*, C^4′^*H*), 4.23 (1 H, m, C^3^*H*), 4.35 (1 H, m, C^11^*H*), 4.53 (1 H, m, C^2′^*H*), 4.60–5.00 (3 H, m, C^15^*H*, C^17^*H*, C^19^*H*), 5.05 (1 H, m, C^1′^*H*), 5.27 (1 H, m, C^33^*H*), 5.88 (1 H, m, C^37^*H*), 5.93–6.38 (13 H, m, C^20–32^*H*_13_).

### 2.6. Repeated Extraction

The polymer was regenerated using water, ethyl acetate and methanol sequentially. In total, 55 cm^3^ of partially purified MeAmB (219 µg cm^−3^, 19%) was passed through the column in three volumes, resulting in a total target retention of 9.88 mg (82%). A total of 187 µg cm^−3^ (85%) was retained from the first volume, 184 µg cm^−3^ (84%) from the second and 176 µg cm^−3^ (80%) from the third. The polymer was then washed with deionised water and a 5 M aqueous solution of ammonium formate. The polymer was then washed again with a small amount of water, followed by diethyl ether, ethyl acetate and dichloromethane. The washing process was completed with a 1:3 methanol–water mixture and the product was eluted with a 1 M methanol solution of ammonium formate, followed by a 2 M solution.

The first 1 M ammonium formate methanol eluate was observed using spectrophotometry to contain approximately 8.8 mg (89.3%) of MeAmB. The second contained 76 µg (0.8%) and the third (2 M) contained 57 µg (0.6%). The ether wash removed 5.8 mg of mainly impurities. The ethyl acetate wash removed 3.2 mg of material, including 58 µg of MeAmB. The dichloromethane eluate contained 10.5 mg of impurities and the methanol–water mix removed 13.5 mg, including a small amount of MeAmB. A total of 2 cm^3^ of water was added to the dry product, which was then sonicated and filtered. The aqueous extract contained 14.4 µg of MeAmB. The residue was collected and lyophilised, giving a total mass of 30 mg.

## 3. Results

### 3.1. General Procedure and Initial Observations

Molecular modelling was used to select appropriate monomers for the synthesis of a biomimetic polymer, with the aim of selectively binding the amphotericin B analogue MeAmB. The basic procedure applied has been used previously in the design of molecularly imprinted polymer synthetic receptors [9,10]. The results gave a series of monomers ranked according to their binding to the target MeAmB. Optimal monomer combinations were determined, and their polymer equivalents were synthesised, according to these results.

Ethylene glycol dimethacrylate (EGDMA) was identified as the optimal functional cross-linker, giving high specificity and affinity for the MeAmB macrocycle, and so was included in most of the studied polymers. Binding of the aminic monosaccharide (mycosamine) required a specific functionality, and a series of eight specific combinations of monomers were trialed empirically based on the results obtained in silico.

Of the eight polymers prepared (shown in Table 1 with the mean target retention), the most promising polymer in the initial examination was a combination of EGMP (ethylene glycol methacrylate phosphate) and EGDMA (ethylene glycol dimethacrylate), labelled as polymer A. Studies with this polymer, however, suggested that releasing the bound MeAmB may be difficult. Ethyl acetate, a common solvent used to regenerate the polymer by removing the remaining material, removed a total of 0.3 µg (3% of column contents after methanol, 1% starting sample). In total, 6.2 µg of MeAmB remained bound to the polymer, equivalent to 62% of the original starting sample, or 91% of what remained following washing and attempted extraction. Polymer A was therefore rejected on the basis that elution of the product may cause difficulties, with the additional concern that the MeAmB may be adversely affected by the relatively acidic environment of the polymer.

**Figure 2:**
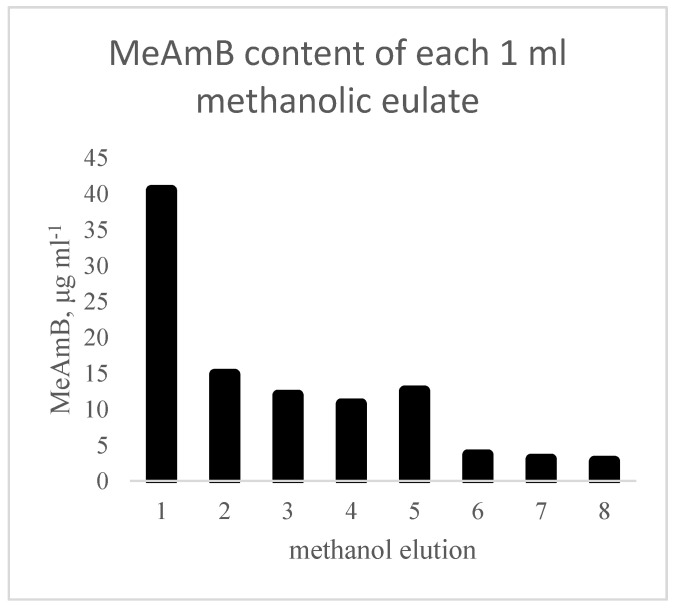
MeAmB content of each 1 mL methanolic eluate after passing through the loaded polymer.

Progression of the research, therefore, transferred to polymer H, prepared from 10% TFMA + 90% EGDMA (polymer H), which was more suitable. Trials with methanol elution showed that methanol alone was sufficient to remove a small proportion of the bound MeAmB, 100 μg (38%) from an average initial loaded content of 262 μg, after eight methanol washes (Figure 2). This was considered to be more appropriate and the 10% TFMA + 90% EGDMA polymer was used for the remainder of the study.

In the general experimental setup, a column was first partially filled with ground polymer and connected to a vacuum chamber. A diagram is provided in Supplementary Figure S1. The impure sample was then added to the column and the target molecule was bound to the functionalised polymer particles. Impurities within the sample with very little affinity for the polymer passed through the column as part of this initial volume and were collected in a vessel within the chamber. The polymer was then washed with various solvents and solutions to remove the remaining impurities, which had some affinity for the polymer or were simply trapped within the column after the initial polymer loading. The target molecule could then be removed from the polymer using a medium for which it had a higher affinity than the polymer.

The requirement, therefore, was the identification of an appropriate composition for the polymer. The MeAmB molecule was reconstructed in silico and screened against a database of functional monomers for optimal binding interactions and locations. A reverse ligand design protocol was adopted to generate a model synthetic binding site for the MeAmB analyte. The eight polymer combinations predicted to generate the most reliable biomimetic polymer were synthesised and evaluated. On analysing both the binding capacity of the polymers and the ease of removing the MeAmB product, a TFMA functionalised polymer was selected. Use of an EGMP polymer was also discussed, but difficulties in removing the bound MeAmB and the possibility of adverse reactions occurring between the polymer and the target led to this being rejected.

### 3.2. Washing of Bacterial Debris

**Figure 3:**
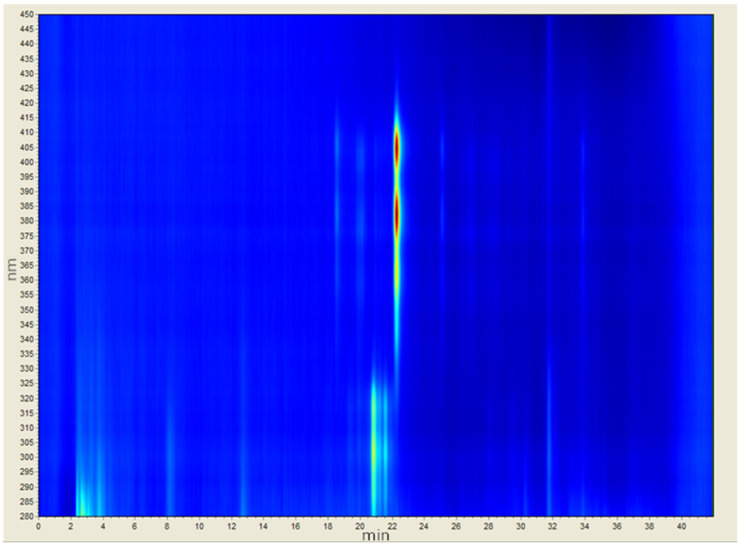
Crude extract. MeAmB can be seen at approximately 22.5 min, but heptaene absorptions of the same wavelengths can be seen earlier and later than this. Tetraenes appear at a shorter wavelength and 21–22 min.

Analytical spectrophotometry-coupled HPLC can be used to demonstrate some of the key considerations in extracting the target (Figure 3). MeAmB is a heptaene, showing a strong response at approximately 22.5 min. MeAmA is a tetraene, and would be found in much lower concentrations than MeAmB in *S. nodosus* NM. MeAmA appears with other tetraenes at a similar time to MeAmB (21–22 min) but at a shorter wavelength in the chromatogram. There are also numerous other polyketides which absorb at similar wavelengths to MeAmB but are separated by the HPLC technique. These are effectively invisible as impurities using standard spectrophotometry due to their similarity to MeAmB, and are only visible using HPLC due to their occurrence at a different time in the spectrum.

In addition to these compounds are the major cell debris components of the crude extract. Carbohydrates made up a large part of the impurities in the initial sample, and can be observed in the range 2–4 min in Figure 3. Lipids were the other major component but were less visible on the chromatogram.

It was quickly determined that aqueous washes could be used to remove a large proportion of the retained carbohydrates without affecting the bound MeAmB. Deionised water was visibly better at removing impurities than aqueous ammonium formate, with water washes producing coloured eluate (impurities) even after buffer solution washes returned colourless. Dichloromethane, similarly, removed large amounts of cell debris, principally carbohydrates, without showing any evidence of removing MeAmB. However, while no obvious damage was observed to this polymer, dichloromethane is corrosive to some plastics, and so some concerns were raised about the use of this solvent. While it has been used previously for similar purposes [11,12], alternatives to dichloromethane were pursued. Simple deionised water was less efficient but effective for the removal of carbohydrates.

Initial results showed that ethyl acetate was greatly superior to diethyl ether in terms of the mass of impurities removed. Diethyl ether may have been the most selective solvent for the extraction of MeAmA (tetraene) from the polymer, but this was not a priority, as MeAmA appeared to be removed as part of the standard bacterial debris removal. It was concluded, therefore, that ethyl acetate was more suitable for the process of washing lipid debris from the polymer, while water was good for sugars (Figure S3) and dichloromethane was generally effective at removing impurities, including carbohydrates left by the water wash (Figure S7).

### 3.3. Target Retention and Elution

**Figure 4:**
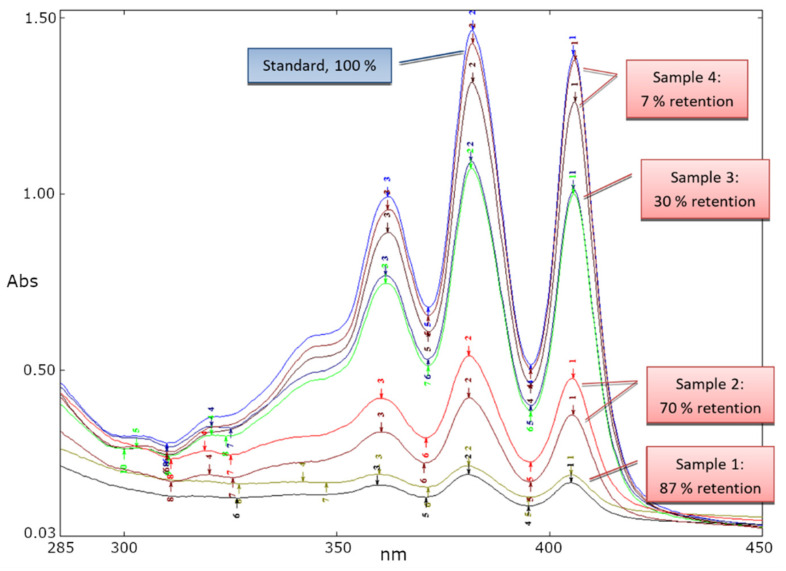
Absorption spectra of MeAmB not retained by the polymer. Measurement was made on the sample after being passed through the MeAmB-specific polymer.

In a study of polymer capacity for MeAmB, two 100 mg polymer columns prepared with a 72 µg cm^−3^ MeAmB sample. The samples were passed through the column in 1 mL volumes and collected after passing through the columns. The spectra of the MeAmB in each 1 cm^3^ sample after passing through the polymer are overlayed below (Figure 4). In the first eluent (Sample 1) 87% of the MeAmB was retained by the polymer. By the fourth sample, an average of just 7% was retained. An average of 138.7 µg was retained on each 100 mg of polymer.

Studies were conducted into removing the product MeAmB from the product with DMSO. Four eluents of 1 mL were found to remove 52% of the bound product, with ten eluents giving 82%. It was established, therefore, that the strength of the binding necessitated a form of ion exchange, as simple solvent affinity could not overcome the MeAmB–polymer bonds.

Aqueous ammonium formate was not found to displace MeAmB or any impurities, but methanolic ammonium formate very effectively eluted the product. This was greatly superior to any solvent observed for this purpose, releasing the vast majority of the bound product in a minimal quantity of solution. This approach did, however, result in ammonium formate in the product sample, which was not easily removed. The optimal concentration was determined to be 0.2 M, as weaker solutions required greater volumes to elute the MeAmB, while stronger solutions gave no benefit but increased residual salt. Ammonium formate will sublime under a strong vacuum, and attempts were made to remove it using this method, though the complexation of the salt and the time required made this unfavourable for further development [13,14].

It was found that a suspension was formed when the dry product of the ammonium formate extraction was added to a minimal amount of water. The aqueous solubility of ammonium formate allowed this to dissolve, leaving the MeAmB with any remaining bacterial impurities. It was found that only 0.2% of the MeAmB content was lost to the aqueous phase with this technique, making it a highly effective method of removing the majority of the residual salt.

The polymer was found to selectively bind to MeAmB over MeAmA, with MeAmB being disproportionately retained on the polymer, and MeAmA being easily removed with common solvents. The results of this process are shown in Table 2.

The results show a selectivity of the column for the amphotericin B analogue MeAmB over the chemically similar co-metabolite 8-deoxy-16-descarboxyl-16-methyl-amphotericin A (MeAmA). This is presumed to be related to the extended linear configuration of MeAmB, in contrast to the less restricted and more compact geometry preferred by AmA and its analogues. It was, therefore, demonstrated on a small scale that the polymer could effectively and selectively bind the MeAmB product, retaining the MeAmB while residual bacterial debris were washed. With ammonium formate, the MeAmB could also be removed from the column after this process was completed in a relatively pure form. This protocol was therefore transferred to a larger scale for optimisation.

### 3.4. Large-Scale Polymer Development

A larger column was filled with 3 g of the biomimetic polymer, allowing greater analysis of the various wash eluates and the final product. The polymer was first run through with water, primarily to remove any ammonium formate remaining from the target extraction, followed by ethyl acetate and methanol. This combination should effectively remove all potential contaminants, leaving the polymer regenerated.

The percentage of MeAmB that was bound to the polymer from all experiments was found to be 81 ± 5%, with little correlation between initial concentration and binding ratio. Greater total retentions would likely be achieved with higher concentrations, therefore, but this is difficult with methanolic solutions. In a larger-scale (3 g of polymer) experiment, a total retention of MeAmB of 3.3 mg per gram of polymer was determined using a sample of 249 mmol dm^−3^ MeAmB (19% purity). This scale allowed more detailed analysis of the product using proton NMR. Previous research with a similar technique, however, found a polymer capacity of 45 mg per gram of polymer for kukoamine A, a large bioactive polyamide obtained from biological sources [15]. It is possible, therefore, that the polymer capacity for MeAmB could be an order of magnitude greater if a suitable delivery solvent was used in the loading procedure.

**Figure 5:**
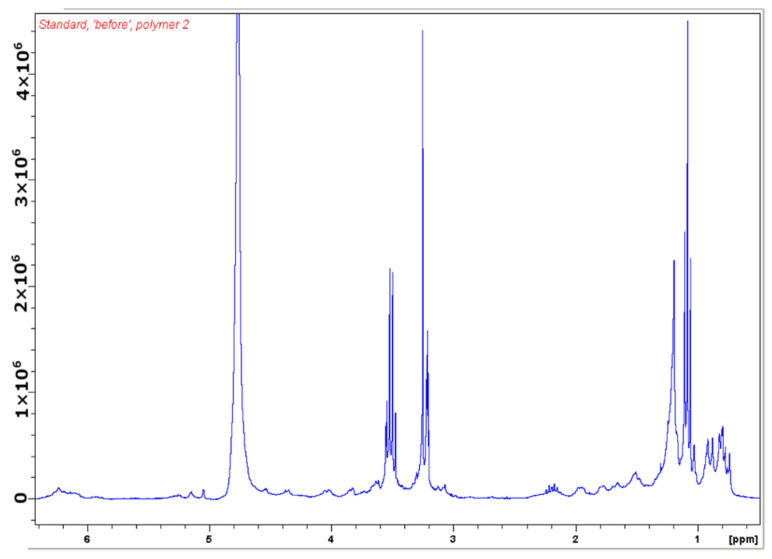
NMR spectrum of the sample loaded onto the polymer. The spectrum shows a small amount of amphotericin B analogue (visible around 6.2 ppm) with greater quantities of carbohydrates, lipids and solvents.

NMR spectroscopy was used to analyse the purification procedure. The spectrum of a typical starting material, a relatively crude bacterial extract, is given below in Figure 5. A small multiplet could be seen at 6.2 ppm, which was the signature resonance of MeAmB and other amphotericin B analogues. Between 3 and 4 ppm sugar frequencies were found, but these were also relatively small in this partially purified extract (the large peaks in this area were solvents). An NMR spectrum principally showing sugar impurities can be found in the supplementary data (Figure S3). The region from 0.5 to 2.5 ppm contained lipids, which were the primary impurity in this sample.

**Figure 6:**
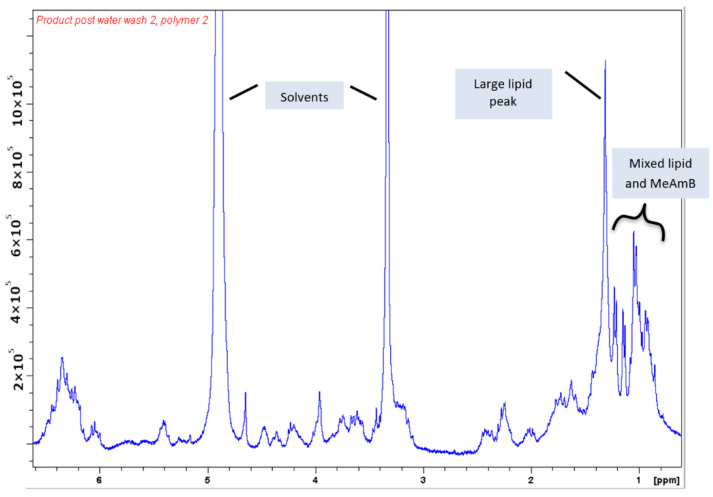
NMR spectrum of the product of polymer purification. The spectrum shows the signature of MeAmB except where highlighted.

After washing, the product was eluted with a methanolic solution of ammonium formate. Removal of the majority of the ammonium formate via dissolution in minimal water and lyophilisation of the precipitate gave the following NMR spectrum (Figure 6).

**Figure 7:**
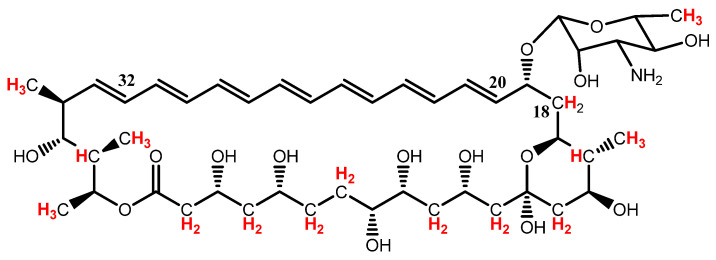
Protons of MeAmB appearing in the 0.5–2.5 ppm region of NMR spectra are highlighted in red. Only one of the carbon-18 protons appears in this region. The protons on carbon-20 to carbon-32 give a characteristic multiplet in the region 6.0–6.6 ppm.

The NMR spectrum produced for the final product shows that the sample contained some residual solvents, along with the product and some impurities. As the mass of the MeAmB product was known, it was possible to approximate the actual purity of the sample in relation to the remaining cell debris. NMR spectra of amphotericin analogues performed in deuterated methanol displayed a characteristic multiplet from 6.0 to 6.6 ppm, arising from the polyene chain of the molecule (protons 20 to 32, Figure 7). As this multiplet typically exists isolated from other frequencies that may come from a sample, it was possible to approximate the relative integration of one MeAmB proton.

Using the measured value of one proton, it was possible to determine the relative integration of the MeAmB protons contributing to the signals between 0.5 and 2.5 ppm, which contained the frequencies of the protons shown below (note that only one of the protons on carbon-18 appears in this region):

The 0.5–2.5 ppm region of the spectra was, therefore, 48% the result of MeAmB. Impurities in MeAmB samples were mainly lipids and sugars, with the former appearing in this region of the spectra and the latter between 3 and 4 ppm. No sugars could be seen in the spectrum for this product, so the primary bacterial impurity in the sample resulted from lipids. The product also contained some ammonium formate, which had a characteristic peak downfield (Figure S4). From the spectrophotometry calculation of the MeAmB mass, the mass of solvent and ammonium formate in the final sample could be calculated using their relative contribution to the final NMR spectrum. Ignoring residual solvent and ammonium formate, therefore, which can be removed by patient lyophilisation, the MeAmB content of the product was approximately 70%, with the remaining 30% being principally bacterial lipids.

Repeated use of the polymer column on a sample was also observed to be a practical way to increase the purity. Several equivalents of crude sample were partially purified by running through the column and combined to give a higher purity product of 10–20% MeAmB. Samples with low MeAmB content (<1%), however, resulted in low overall yield from the polymer extraction process.

From this, it can be shown that MeAmB samples of 70% can be generated in a single use of the biomimetic polymer from a sample of 19% MeAmB, with successful removal of almost all bacterial debris. Samples of 19% or more can easily be produced from running relatively crude samples of approximately 1%, and samples of higher quality than 70% should be possible, if required, with repeated use of the column.

A general protocol for the extraction of MeAmB can, then, be outlined as follows. Firstly, the compound can be extracted from the raw bacterial matrix with a simple solvent extraction process, bringing the concentration to approximately 1%. From here, the polymer can be used, firstly to bring that 1% to approximately 20%, then in a repeat to approximately 70%. A possible third step would then be the purification of this 70% using HPLC. Each process is optimal for the quality of the sample, in that the efficacy of solvent extraction techniques diminishes above 1%, but the polymer functions best at 1% or higher concentrations. Use of the biomimetic polymer described herein also becomes inefficient above 70%, leading to the loss of product and increasing salt content. Finally, HPLC is an effective method of purifying samples, but requires samples of relatively high purity (i.e., 70%) to avoid blocking the column with bacterial lipids.

## 4. Conclusions

Amphotericin B has been an essential component in the treatment of life-threating diseases for decades. Its toxicity, however, has demanded the development of improved analogues. 16-descarboxyl-16-methyl amphotericin B (MeAmB) is amongst the best of these, but the difficulty found in isolating and purifying MeAmB has restricted its medicinal use. With the application of biomimetic polymers, this may no longer be an issue. MeAmB has been successfully taken directly from its biological source and, in a few simple procedures, has been qualitatively enhanced beyond that which could be achieved using traditional methods, while using a fraction of the time and resources. This should provide a more efficient process for the production of high-quality drugs obtained from biological sources.

## Figures and Tables

**Table 1:** Mean retention of 10.1 µg cm^−3^ (A–D) and 10.0 µg cm^−3^ (E–H) MeAmB sample by eight different polymers analysed in duplicate. Abbreviations: EGMP, ethylene glycol methacrylate phosphate; EGDMA, ethylene glycol dimethacrylate; AMPSA, 2-acrylamido-2-methyl-1-propanesulfonic acid; DEIA, desethyl-desisopropyl atrazine; IA, itaconic acid; MAA, methacrylic acid; TFMA, 2,2,2-Trifluoroethyl methacrylate.

Label	Composition	Mean MeAmB Retention	
A	5% EGMP + 95% EGDMA	8.0 µg cm^−3^	79%
B	20% EGMP + 80% EGDMA	7.3 µg cm^−3^	72%
C	10% EGMP + 1% AMPSA + 89% EGDMA	7.6 µg cm^−3^	75%
D	30% AMPSA + 70% EGDMA	6.5 µg cm^−3^	65%
E	10% DEIA + 90% EGDMA	4.6 µg cm^−3^	46%
F	10% IA + 90% EGDMA	5.4 µg cm^−3^	54%
G	20% MAA + 80% ECOMA	3.9 µg cm^−3^	39%
H	10% TFMA + 90% EGDMA	7.4 µg cm^−3^	74%

**Table 2:** Average results of passing four separate samples through the polymer three times in an analysis of selectivity for MeAmB over tetraene side product MeAmA.

Sample	MeAmB Retained	Tetraene Retained
1	63 µg (58%)	47 µg (47%)
2	38 µg (35%)	14 µg (14%)
3	13 µg (12%)	−4 µg (−4%)
4	15 µg (14%)	2 µg (2%)
**Total**	**128.3 µg**	**59.2 µg**

## Data Availability

Data is available on request.

## Supplementary Materials

The following supporting information can be downloaded at: https://www.mdpi.com/article/10.3390/biomimetics8030273/s1, Figure S1: Diagram of the polymer-filled fritted column (top) and the vacuum chamber used in the extraction (bottom); Figure S2: Structure of amphotericin B with numerical identifiers to proton assignment; Table S1: Proton resonance of amphotericin B (as determined by Sowiński et al.) with identifiers corresponding to those in Figure S2; Figure S3: Water wash eluate. Sugars can be seen in the region between 3 and 4 ppm, and lipid around 1 ppm. No MeAmB is obviously present, the characteristic multiplet of which occurs around 6.2 ppm; Figure S4: Sample after a single run through the polymer, showing bacterial debris, solvents and ammonium formate at 8.4 ppm; Figure S5: NMR spectrum of the product after rerunning through the polymer with simple water and ethyl acetate washes to remove the impurities; Figure S6: HPLC UV-Vis chromatogram for a crude methanolic extract; Figure S7: HPLC chromatogram of sugars in the dichloromethane eluates.
