# Supplementary material for: A Biomimetic Polymer for the Extraction and Purification of Superior Analogues of Amphotericin B
Source: Biomimetics (Basel). 2023 Jun 27;8(3):273. doi: 10.3390/biomimetics8030273 (PMC10377485; doi:10.3390/biomimetics8030273)
Supplement: Supplementary file 1 [file biomimetics-08-00273-s001.zip › biomimetics-2458310-supplementary.pdf]

Supplementary information - A biomimetic polymer for the extraction and purification of superior analogues of amphotericin B

S1. Basic apparatus

The polymer columns (Figure S1, top) were connected to a vacuum chamber (Figure S1, bottom) via rotatable inlets (eight per chamber) which allow control of the vacuum experienced by the contents of the column. All liquids were run into a collection vessel within the chamber at a rate no greater than 1 drop per second.

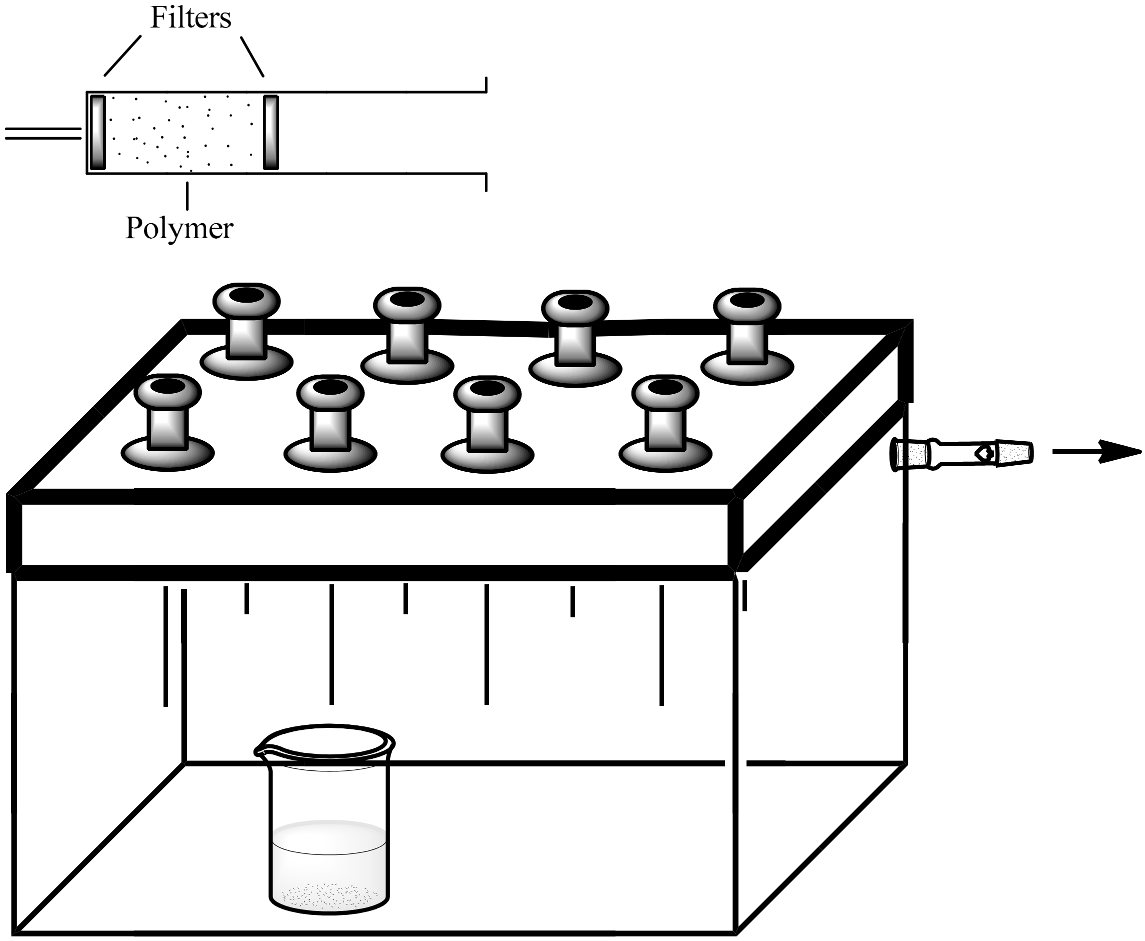

Figure S1. *Diagram of a polymer-filled fritted column (top) and the vacuum chamber used in the extraction (bottom).*

S2. NMR analysis background

Comparative analysis of wild-type amphotericin B in previous research has provided information on the resonant frequencies of the AmB protons.[i] Due to the size of the molecule and the complexity of the spectra resulting from its examination, combined with the variable purity of the samples observed, this information was essential to understanding the spectra produced during this project.

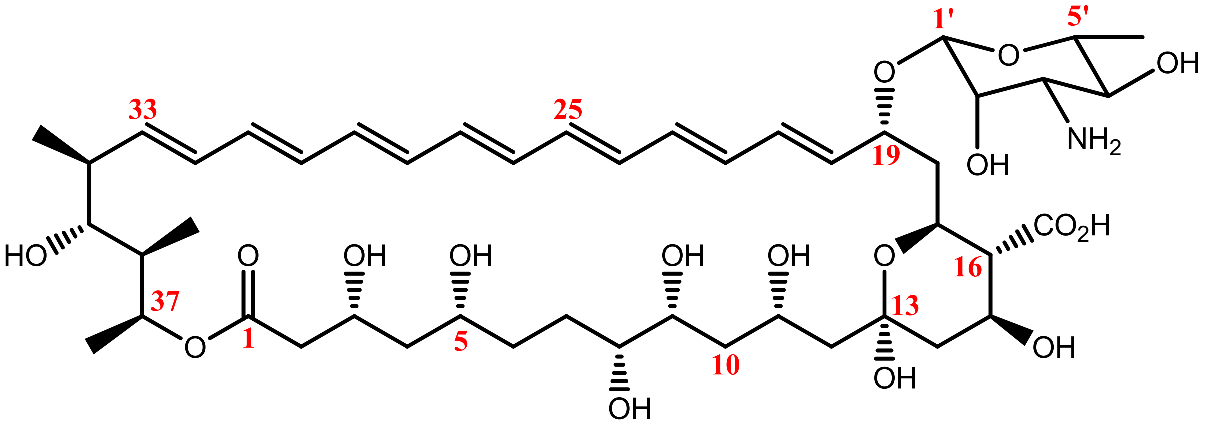

Figure S2. *Structure of amphotericin B with numerical identifiers to proton assignment.*

^{1}H	δ, ppm	^{1}H	δ, ppm	^{1}H	δ, ppm	^{1}H	δ, ppm
**2**	2.65; 2.48	**11**	4.84	**21 – 32**	6.3 – 6.8	**1'**	5.38
**3**	4.69	**12**	1.95; 1.72	**33**	5.56	**2'**	4.95
**4**	1.86; 1.65	**14**	2.53; 1.75	**34**	2.71	**3'**	3.93
**5**	4.16	**15**	5.04	**35**	3.45	**4'**	4.29
**6**	2.01; 1.73	**16**	2.85	**36**	2.13	**5'**	4.06
**7**	2.45; 1.83	**17**	5.23	**37**	5.85	**5' – Me**	1.62
**8**	3.64	**18**	3.10; 2.03	**34 – Me**	1.33		
**9**	4.07	**19**	5.01	**36 – Me**	1.27		
**10**	2.22; 1.56	**20**	6.59	**37 – Me**	1.48		

Table S1. *Proton resonance of amphotericin B (as determined by Sowiński et al.[i]) with identifiers corresponding to those in Figure S2.*

S3. NMR analysis of cell debris

The proton NMR spectrum of the product resulting from washing the loaded column with water is presented below (Figure S3). The spectrum shows primarily carbohydrates, with a large characteristic signal at 3-4 ppm. No MeAmB can be observed, which gives a characteristic multiplet above 6 ppm.

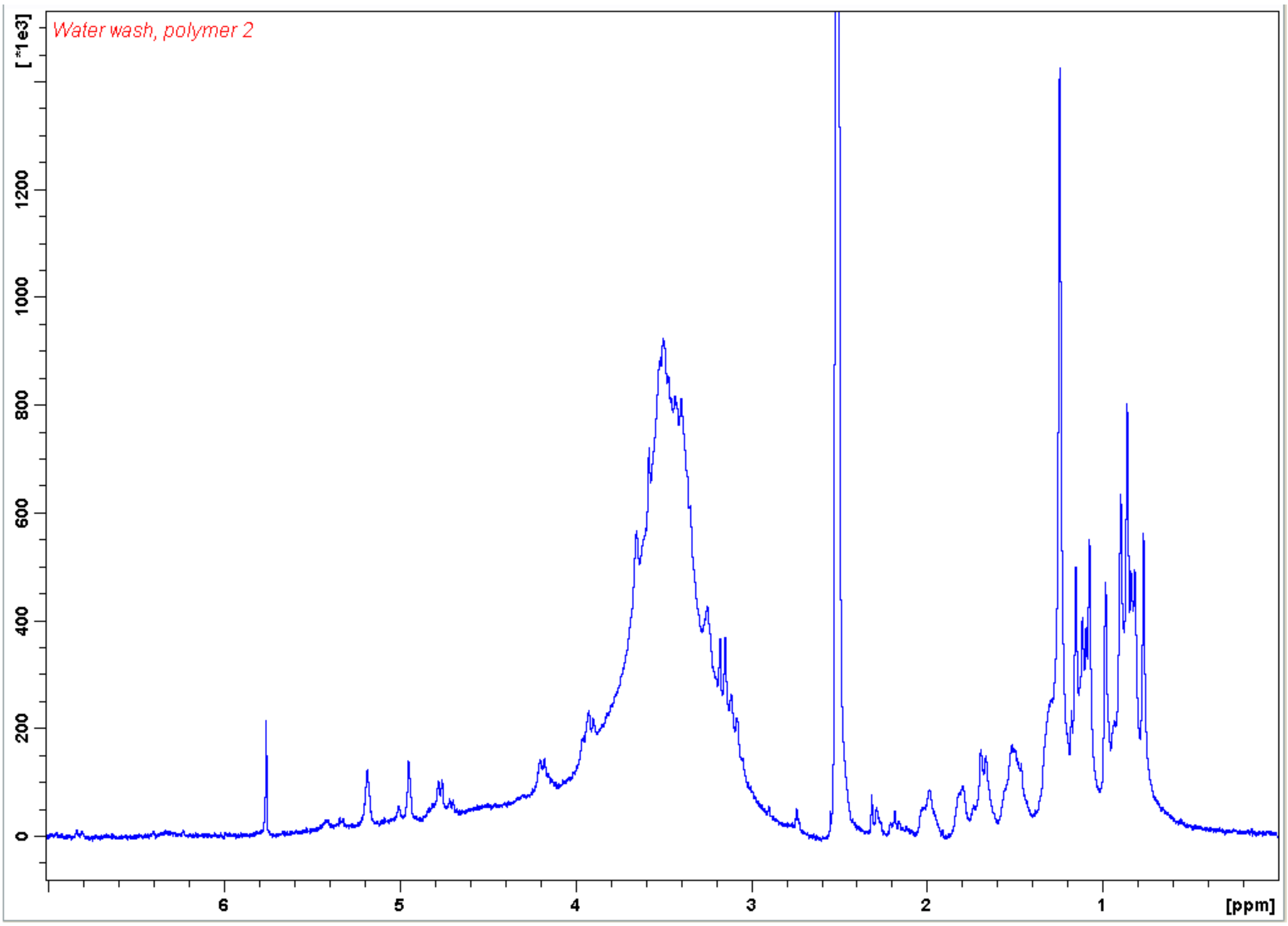

Figure S3. *Water wash eluate. Sugars can be seen in the region between 3 and 4 ppm, and lipid around 1 ppm. No MeAmB is obviously present, the characteristic multiplet of which occurs around 6.2 ppm.*

S4. Effect of rerunning samples

NMR spectra show a sample before and after a second run through the polymer, including simple washes with water and ethyl acetate. The spectra for the sample before passing through the polymer (Figure S4) a second time shows evidence of some residual impurities from the bacterial matrix, notably the peak at approximately 1 ppm corresponding to certain lipids, and sugars in the region 3-4 ppm.

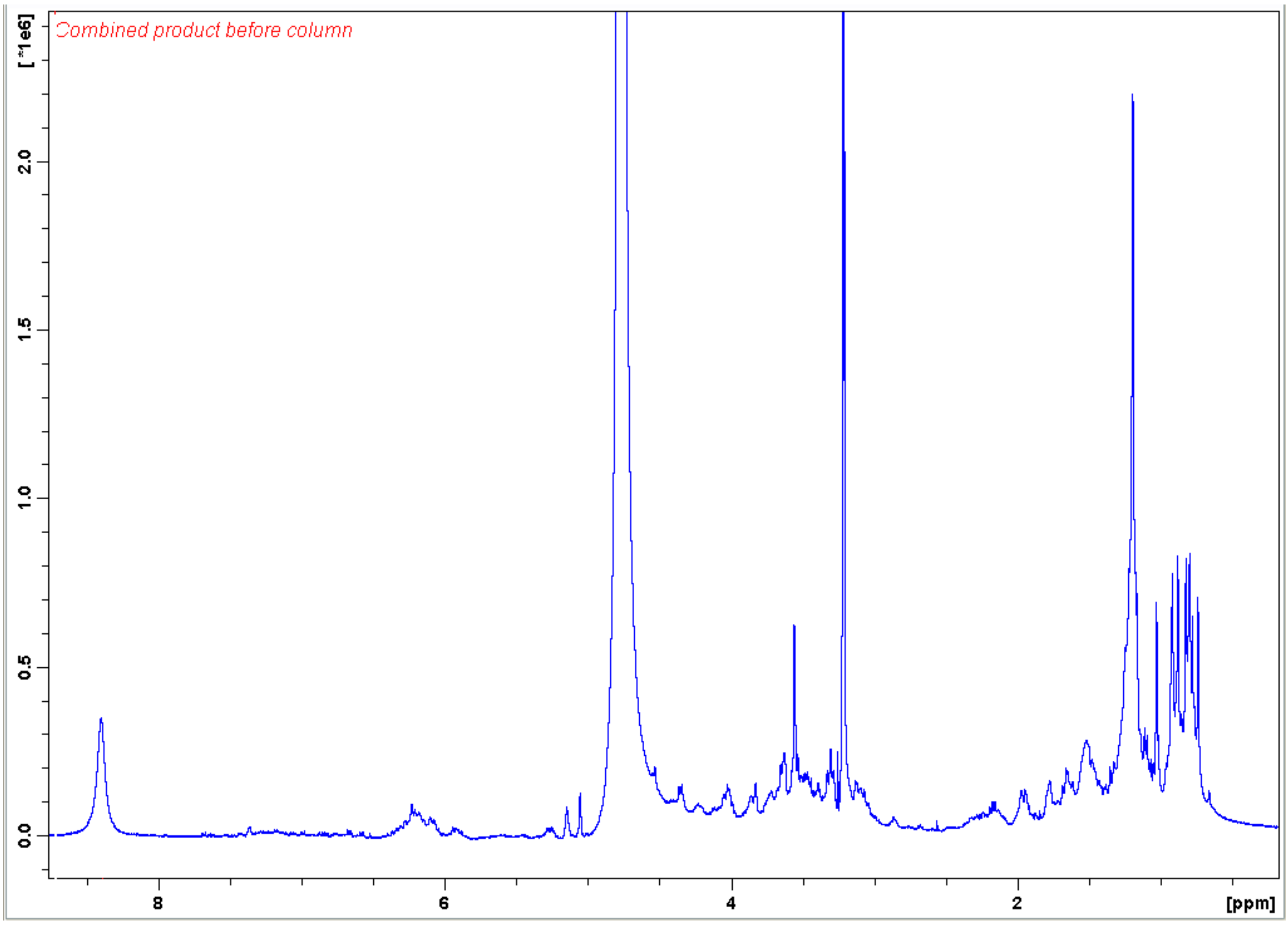

Figure S4. *Sample after a single run through the polymer, showing bacterial debris, solvents and ammonium formate at 8.4 ppm.*

The purity is improved in the rerunning process, as shown in second NMR spectrum (Figure XXX). The amphotericin B associated multiple at 6.2 ppm is relatively larger, showing than some of the bacterial debris has been removed.

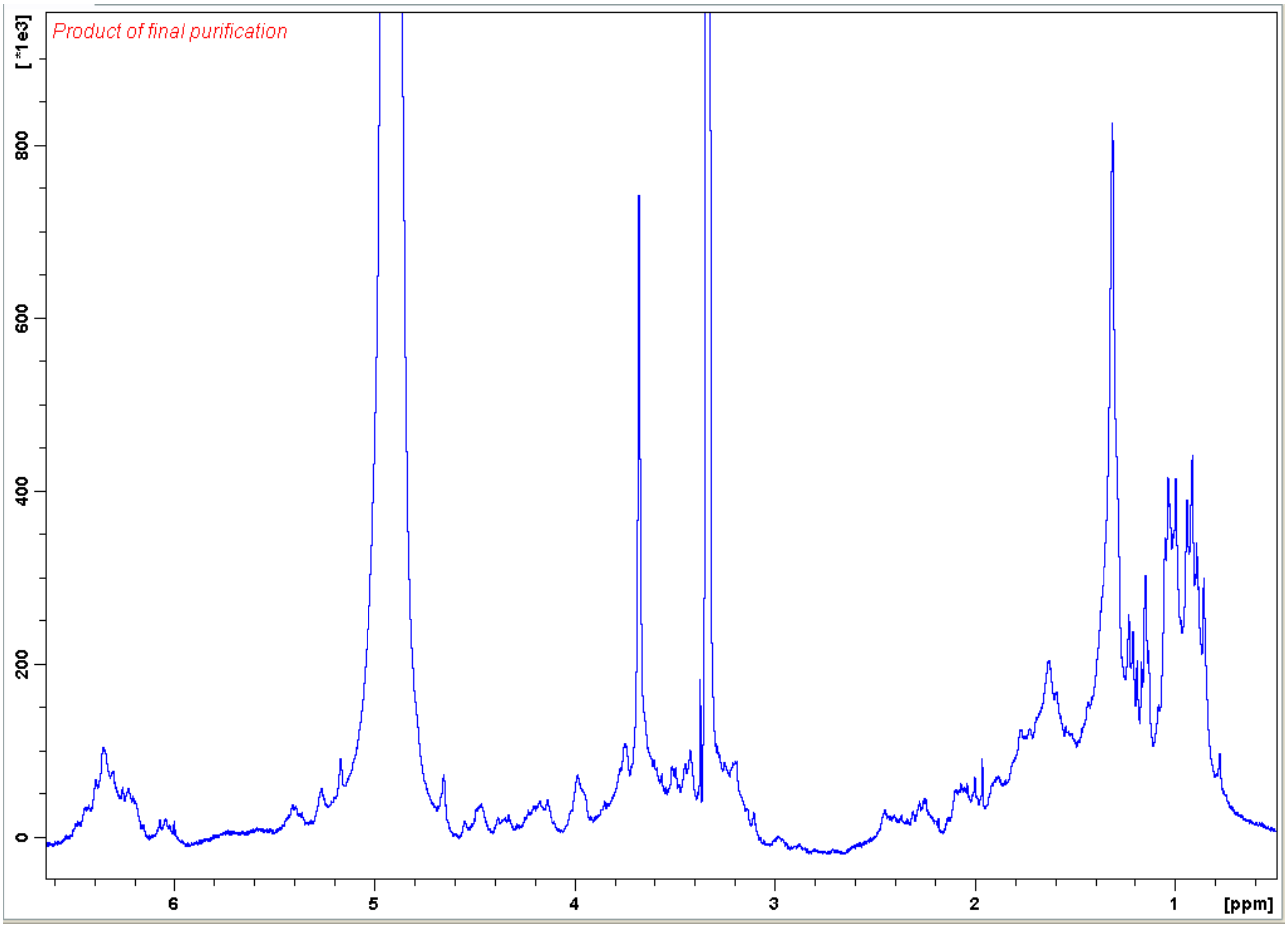

Figure S5. *NMR spectrum of the product after rerunning through the polymer with simple water and ethyl acetate washes to remove the impurities.*

S5. Spectrophotometry coupled HPLC

The major technique for the standard analysis and purification of amphotericin B and its variants is HPLC. However, HPLC is not suited to raw bacterial extract, and requires the samples to be somewhat purified prior to its use for purification. Spectrophotometry coupled HPLC can be used to give a chromatogram of a relatively crude bacterial extract as a reference (Figure S6).

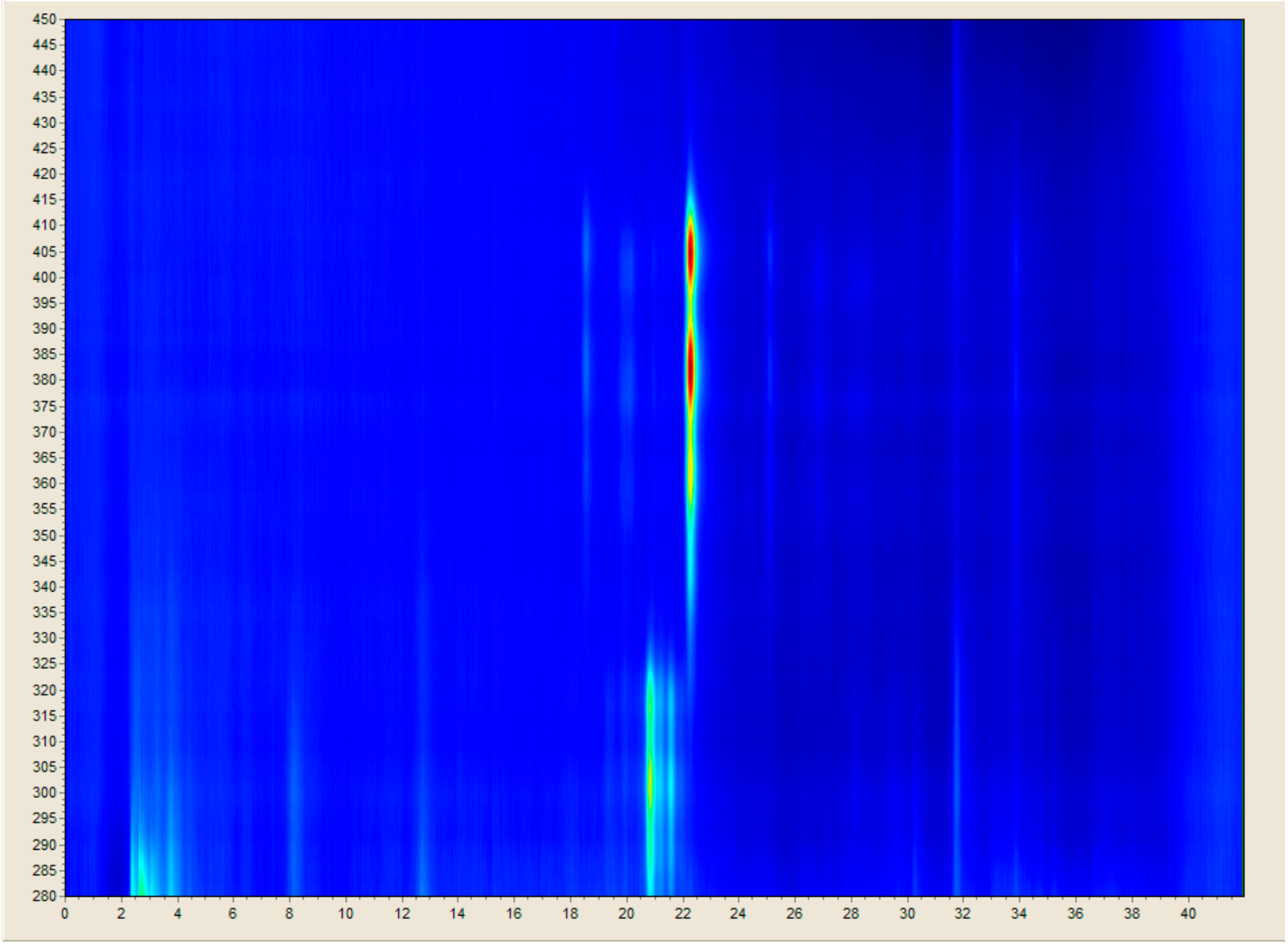

Figure S6. *HPLC UV-Vis chromatogram for a crude methanolic extract.*

The three strong absorption peaks of MeAmB, at 405 nm, 380 nm and 360 nm, can be seen at approximately 22 minutes. The shorter wavelength tetraene (amphotericin A) absorption can be seen just before this, and some impurities can also be observed by this method, for example the sugars at just after 2 minutes. Many impurities are not observable with this technique.

The use of reverse phase chromatography with an increasingly non-polar mobile phase as time passes ensures that more hydrophilic compounds injected into the apparatus are displayed first in the chromatogram. This can be demonstrated with a sample of mostly

carbohydrates which were washed from the loaded polymer with dichloromethane, Figure S7, which gives a response at just over 2 minutes.

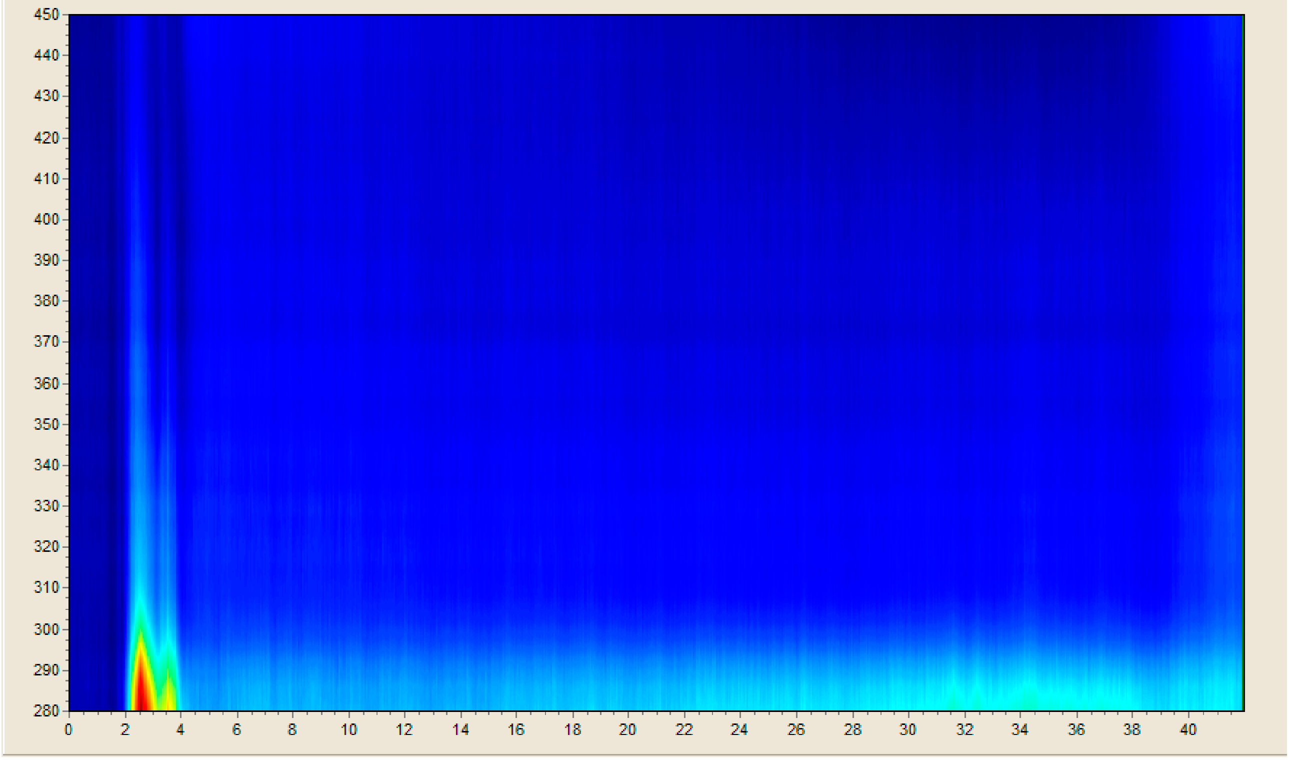

Figure S7. *HPLC chromatogram of sugars in the dichloromethane eluates.*

---

[i] P. Sowiński, J. Pawlak, E. Borowski, *Magn. Reson. Chem.*, 1992, **30**, 275-279.
